# Recent advances in understanding Kaposi’s sarcoma-associated herpesvirus

**DOI:** 10.12688/f1000research.7612.1

**Published:** 2016-04-25

**Authors:** Nathan J. Dissinger, Blossom Damania

**Affiliations:** 1 Lineberger Comprehensive Cancer Center and Department of Microbiology & Immunology, University of North Carolina at Chapel Hill, Chapel Hill, NC, USA

**Keywords:** Kaposi’s sarcoma-associated herpesvirus, KSHV, primary effusion lymphoma, multicentric Castleman’s disease

## Abstract

Kaposi’s sarcoma (KS)-associated herpesvirus (KSHV) is an oncogenic human herpesvirus. KSHV is associated with three cancers in the human population: KS, primary effusion lymphoma (PEL), and multicentric Castleman’s disease (MCD). KS is the leading cause of cancer in HIV-infected individuals. In this review, we discuss the most recent discoveries behind the mechanisms of KSHV latency maintenance and lytic replication. We also review current therapies for KSHV-associated cancers.

## Introduction

Kaposi’s sarcoma (KS)-associated herpesvirus (KSHV), also known as human herpesvirus 8 (HHV-8), is a linear double-stranded DNA virus and a member of the gammaherpesvirus subfamily. The virus was first isolated by Chang *et al.* in KS biopsy samples from AIDS patients ^1^. Subsequent studies further identified KSHV as the etiologic agent of primary effusion lymphoma (PEL) ^2^ and the B-cell hyperplasia known as multicentric Castleman’s disease (MCD) ^3^. KSHV is also linked to two under-studied inflammatory syndromes. One KSHV inflammatory disease recognized, immune reconstitution inflammatory syndrome-KS (IRIS-KS), is the paradoxical rapid development of KS after the start of highly active antiretroviral therapy (HAART) for HIV and during the rebound of CD4+ T-cells ^4, 5^. Uldrick *et al.* discovered another inflammatory disease, termed KSHV inflammatory cytokine syndrome (KICS), in patients infected with both HIV and KSHV with high levels of viral interleukin-6 (vIL-6), human IL-6 (hIL-6), and KSHV viral loads ^6^. Subsequent to this initial report, KICS has also been found to affect non-HIV-infected KSHV-positive individuals ^7^.

KSHV, like other herpesviruses, has a latent and lytic phase to its lifecycle ^8, 9^. Following primary infection, both latent and lytic genes are expressed, but after several rounds of replication, lytic gene expression decreases and latency is established. Latency is the default program of the virus ^10^. During latency establishment, the linear KSHV genome circularizes to become an episome. This latent form of KSHV expresses only a few proteins, including latency-associated nuclear antigen (LANA), viral FADD-like interleukin-1-β-converting enzyme (FLICE/caspase 8)-inhibitory protein (vFLIP), vCyclin, and multiple microRNAs ^8, 11^. Additional genes that are expressed at low levels during latency include K1, vIL-6 ^12^, and K15 ^13^. The expression of LANA is sufficient and necessary to establish latency, as it plays a pivotal role in episome maintenance and latent replication. Two LANA proteins form a dimer and the N-termini bind to the host chromosomes while the C-termini interact with LANA-binding sites (LBSs) in the KSHV episome ^14^. Recently, three labs have crystalized the C-terminus of LANA and found that the LANA dimers oligomerize, forming a higher order of organization that facilitates the binding of DNA ^15– 18^. It was also discovered that LANA contains positively charged patches opposite to the DNA-binding face. Mutations of these residues did not alter LANA’s DNA binding capabilities but diminished the interaction with cellular chromatin bromodomain (BRD) proteins, which play a role in latent replication and maintenance ^16, 17, 19^.

Lytic replication is divided into three phases of gene expression: immediate early (IE), delayed early (DE), and late ^8, 20^. As the transcription of IE genes does not require prior viral protein synthesis, IE genes are experimentally defined by their transcription in the presence of inhibitors of protein synthesis such as cycloheximide. DE gene expression can be inhibited by cycloheximide because they require proteins encoded by IE genes to transactivate their promoters but are also not dependent on DNA replication. Late genes are expressed subsequent to the start of viral DNA replication and encode for structural proteins required for assembling new virions as well as envelope glycoproteins. Viral replication inhibitors (e.g. the viral polymerase inhibitor ganciclovir) can prevent the production of infectious progeny virions.

Latent KSHV can be induced into lytic replication with the addition of chemicals such as 12-O-tetradecanoylphorbol-13-acetate (TPA), valproic acid (VPA), and sodium butyrate. These chemicals activate the expression of the IE gene replication and transcription activator (RTA), encoded by ORF50, which is the key regulator of KSHV lytic replication as its ectopic expression is sufficient to start the lytic cascade ^8^. However, it has recently been proposed that KSHV can be reactivated and enter lytic replication in a RTA-independent manner ^21, 22^. In this pathway, KSHV reactivation is induced by cellular apoptosis and is dependent on the activation of caspase 3. It is interesting to note that the virions produced though this RTA-independent lytic pathway appear to be less infectious than virions produced through RTA-dependent lytic replication ^21^. This observation needs to be furthered expanded upon in the future.

KSHV is a pathogenic virus whose mechanism of disease is not fully understood. It is clear that both the latent and lytic phases of the KSHV lifecycle play a role in virus-related disease and a better understanding of these phases can help guide the development of treatments. This review covers recent advances in understanding the latent/lytic switch and discusses current and potential future therapeutic treatments for KSHV-related malignancies.

## Maintenance of KSHV latency

**Figure 1.**
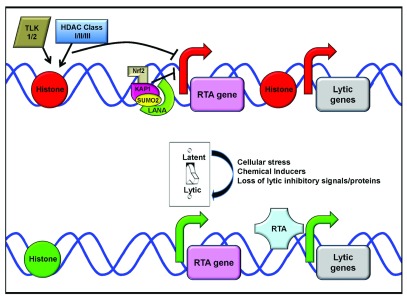
Schematic of Kaposi’s sarcoma-associated herpesvirus (KSHV) latent/lytic switch. During latency, only a few viral proteins and microRNAs are expressed. The KSHV latent protein latency-associated nuclear antigen (LANA) establishes latency and tethers the KSHV episome to host chromosomes. During this phase of the KSHV lifecycle, lytic genes are suppressed. This suppression occurs due to chromatin modifications that put the replication and transcription activator (RTA) gene and other lytic genes in a closed chromatin conformation with histones that contain inhibitory marks (histones shown in red). These inhibitory modifications are likely regulated by histone deacetylases (HDACs) and tousled-like kinases (TLKs). LANA (lime green semi-circle) also suppresses RTA expression through a complex with poly-SUMO-2-ylated KAP1 (pink tear-drop with yellow circle) and nuclear factor E2-related factor 2 (Nrf2) (tan L) that binds to the RTA gene promoter, further inhibiting transcription (indicated by the red arrow). Upon addition of inducers of the latent/lytic switch, e.g. cellular stress or 12-O-tetradecanoylphorbol-13-acetate (TPA), the chromatin around lytic genes is opened. The histones associated with lytic genes lack inhibitory marks and contain activation marks (histones shown in green). This results in gene transcription from the RTA promoter being activated (green arrow), allowing for RTA expression and transactivation of downstream lytic genes.

How latent KSHV reactivates and efficiently makes new progeny virus is a complex process that requires not only viral but also cellular proteins. To maintain latency, it is important that latent genes are expressed while lytic gene transcription is repressed ^23^. The KSHV episome is packaged in chromatin and several labs have shown that in actively transcribed latency regions, the chromatin is in an open configuration, lacks nucleosomes, and exhibits active histone marks while lytic genes are packaged in closed chromatin ( Figure 1) ^24– 30^. Recently, LANA has been found to bind to both viral and cellular transcriptional start sites that contain histones with the active H3K4me3 mark, allowing the packaged DNA to be more accessible and actively transcribed ^31^. Interestingly, LANA was also found to interact with the H3K4 methyltransferase hSET1, indicating LANA’s potential to play an active role in altering epigenetic changes ^31^. Indeed, histone modifiers play an important role in the maintenance of latency ^31^. Class I and class II histone deacetylases (HDACs) have been shown to repress TPA-induced reactivation through epigenetic changes ^32^. Li *et al.* examined the effect of class III HDACs, known as sirtuins (SIRTs), and found that they also repress reactivation through epigenetic changes ^33^. SIRT1 is able to inhibit lytic replication through its ability to bind to RTA and inhibit its transactivation activity ^33^. In fact, inhibition of SIRT1 was sufficient to induce lytic replication ^33^. Dillon *et al.* reported that the knockdown of another family of histone-modifying enzymes, the tousled-like kinases (TLKs), resulted in loss of latency and reactivation of the virus ^34^. This was due to a decrease in inhibitory phospho-histone H3 associated with the RTA promoter.

Besides epigenetic changes, cellular proteins play a role in the maintenance of latency through direct interactions with viral proteins. Krüppel-associated box domain-associated protein 1 (KAP1) is a chromatin remodeler, and several groups have shown that it also interacts with LANA ^35– 37^. Cai *et al.* reported that this interaction is strengthened by poly-SUMO-2-ylation of KAP1 so it can bind to a LANA SUMO-2 interacting motif ^37^. LANA and KAP1 form a complex with another cellular protein named nuclear factor E2-related factor 2 (Nrf2) ^38^, which targets the RTA promoter and allows for LANA-KAP1 to inhibit RTA expression, thereby repressing lytic replication ( Figure 1) ^35, 36^.

Heat shock protein 90 (HSP90) is a cellular chaperone protein that interacts with the N-terminus of LANA ^39^. Using the HSP90 inhibitors 17-dimethylaminoethylamino-17-demethoxygeldana-mycin (17-DMAG) and AUY922, Chen *et al.* disrupted this interaction and found it led to degradation of LANA ^39^. It was also observed that these inhibitors along with a third HSP90 inhibitor (PU-H71) caused apoptosis of PEL cell lines. Another group also reported an increase in apoptosis of PEL cell lines treated with PU-H71 ^40^. K1 is another viral protein involved in preventing apoptosis that was found to interact with HSP90 ^41^. When cells expressing K1 were treated with HSP90 inhibitors, it was discovered that K1 expression was decreased and K1’s anti-apoptotic effect was diminished. These studies show the important role of HSP90 in maintaining latency through stability of LANA ^39^ and inhibition of apoptosis ^39– 41^.

## Efficient lytic replication of KSHV

Once KSHV is reactivated, it is important for efficient completion of the lytic cycle to make infectious viral progeny. Though RTA is the driver of reactivation, completion of the lytic cycle requires cellular proteins. The KSHV IE/DE protein ORF45 has been shown to activate cellular kinases in the ERK-RSK pathway, and inhibition of this leads to reduced lytic replication ^42^. Recently, it has been found that sustained activation of ERK-RSK leads to the phosphorylation and accumulation of c-Fos, which binds to KSHV promoters ^43^. This accumulation of active c-Fos allows for efficient late lytic gene expression, as shown by a knockdown of c-Fos resulting in a decrease of ORF64 lytic gene expression and the fact that a non-functional form of c-Fos resulted in decreased virion production. Fu *et al.* also examined ORF45 activation of ERK-RSK signaling and discovered that amino acids 56–70 of ORF45 are critical for its interaction with RSK ^44^. In fact, a single amino acid mutation of ORF45 at F66 can disrupt its ability to activate RSK, which leads to decreased late lytic gene expression and reduction of new virus. It was also shown that reactive oxygen species (ROS) can induce KSHV reactivation from latency ^45^ and that induction of the KSHV lytic cycle further upregulates ROS, which can be targeted with N-acetyl-L-cysteine (NAC) to inhibit the development of KS ^46^.

Another pathway shown to be important for late lytic replication is the DNA damage response (DDR) pathway. Hollingworth *et al.* have demonstrated that upon reactivation, early lytic gene expression activates DDR kinases ^47^, as does primary infection ^48^. When inhibitors of ATM and ATR were added to cell culture, it was found that the virus could reactivate and enter lytic replication, but late gene expression was diminished, resulting in fewer infectious viral progeny being made ^47^.

## Current therapies

Most KSHV-infected cells harbor the latent form of the virus. In the case of KS and PEL, most tumor cells are latent with only a few cells exhibiting lytic gene expression. In MCD, a larger proportion of the tumor mass displays lytic gene expression. Lytic replication is thought to be required to promote the growth of KSHV-associated cancers and help spread the virus. In most cases, the high proportion of cells undergoing abortive lytic replication express lytic proteins involved in the activation of angiogenesis and signal transduction, and complete viral replication does not occur ^9^. Some researchers have hypothesized that the induction of lytic replication would be a good therapy for KSHV cancers if used in combination with a lytic inhibitor such as ganciclovir. By initiating reactivation but not allowing full lytic replication, more immunodominant targets could be produced that would be recognized by the immune system and provide more druggable targets to kill infected cells ^49, 50^.

In 2011, a pilot study was published in support of induction therapy in the treatment of KSHV-related MCD ^51^. In this study, patients were treated with high-dose zidovudine along with valganciclovir. The KSHV kinases ORF36 and ORF21 phosphorylated these compounds, making them toxic to the cell. Overall, 86% of treated patients obtained a major clinical response and 50% obtained a major biochemical response as determined by improvements in clinical parameters such as hemoglobin, albumin, and C-reactive protein levels. The 5-year survival rate reported in this study was 86% ^51^. Another report showed that *in vitro* treatment of KSHV-infected cells with the HIV protease drug nelfinavir resulted in less infectious KSHV virus being produced ^52^.

In a search for effective inducers of lytic replication, Kang *et al.* screened 650 US Food and Drug Administration (FDA)-approved drugs in an *in vitro* assay ^53^. This screen identified three topoisomerase II inhibitors (doxorubicin, daunorubicin, and epirubicin) as strong inducers of viral reactivation and that daunorubicin was even more powerful than the classic inducer, sodium butyrate. These three drugs were able to cause apoptosis through DNA intercalation, but the virus produced was capable of infecting new cells. Hence, if these inducers were to be used in patients, it would require their use in combination with a viral replication inhibitor.

Several groups have shown that latency is linked to a dysregulated metabolic state of the cell with increased fatty acid synthesis ^54– 56^. SIRT1 function is also linked to promoting increased fatty acid synthesis, and, as previously stated, inhibition of SIRT1 leads to increased lytic replication ^33^. Bhatt *et al.* showed that inhibition of fatty acid synthase (FASN) with a drug, C75, led to cellular apoptosis by activation of caspase 3 (another inducer of lytic replication, as discussed above) ^54^. Moreover, KSHV-latent endothelial cells go through caspase 3/7-induced apoptosis when glutaminolysis is inhibited ^57^. Dai *et al.* have also demonstrated that by inhibiting sphingosine kinase 2 and sphingolipid metabolism, PEL cells build up ceramides in the cell that result in lytic replication and apoptosis ^58^. Furthermore, Leung *et al.* also demonstrated that clinically achievable amounts of the glucose metabolic analog 2-Deoxy-D-glucose (2-DG) induce endoplasmic reticulum stress and inhibit KSHV replication and reactivation from latency ^59^. These data suggest that new therapeutics targeting metabolic pathways in KSHV cancer cells should also be explored.

Other modes of therapies for KSHV-associated cancers have also been reported. Valiya Veettil *et al.* recently reported that latent KSHV cells have increased glutamate secretion and metabotropic glutamate receptor 1 expression ^60^. Inhibitors of glutamate secretion and receptor expression in KS and PEL cells were found to decrease cellular proliferation. Another study has demonstrated the ability of the drug celecoxib to suppress RTA expression and viral production by blocking the activation of the p38 MAPK pathway ^61^. Another key pathway that has been targeted is the PI3K/Akt/mTOR pathway. Sin *et al.* demonstrated that the use of the mTOR inhibitor rapamycin was capable of inhibiting PEL tumor growth by reducing cytokine secretion and autocrine signaling ^62^. Since then, more reports have come out showing other inhibitors of this pathway have similar effects ^63– 65^. It is interesting to note that not only does rapamycin inhibit tumor growth but it is also capable of inhibiting viral reactivation by repressing RTA expression through transcriptional and post-transcriptional mechanisms ^66^.

Another pathway, the Notch signaling pathway, which is activated by KSHV, has recently been reported to cause endothelial-to-mesenchymal transition (EndMT) ^67, 68^ through the upregulation of membrane-type 1 matrix metalloproteinase (MT1-MMP) ^67^ and the transcription factors Snail, Slug, Twist, ZEB1, and ZEB2 ^68^. This EndMT event allows for the KSHV-infected cell to invade other tissue, an important aspect for the development of KS, and this knowledge of Notch signaling and KSHV provides new molecular targets for therapy.

## Concluding thoughts

KSHV is a double-stranded DNA oncogenic herpesvirus. After infection, the virus goes latent and expresses only a few proteins and microRNAs. This latent virus can be reactivated and enter the lytic cycle through either cellular stress or chemical induction that alters the epigenetics of the cell. During the complete lytic cycle, the virus expresses its genes in a temporal fashion and produces new, infectious virus particles that ultimately kill the cell.

Even though the lytic cycle is important for pathogenesis, the vast majority of cells in KSHV malignancies harbor latent virus. Viral induction therapy is a promising method to treat KSHV-related diseases. It is important, however, to create a balance between efficient reactivation of latent cells and controlling the spread of infection through the use of combination therapies involving lytic replication inhibitors. This method of treatment has the potential to induce immunodominant viral proteins, cause apoptosis of the cell, and inhibit the production of structural proteins so new virions cannot be made. Future experiments should explore new combinations of KSHV-reactivating drugs and late lytic cycle inhibitors. Some potential inducers to be used in these experiments include the anthracyclines and HSP90 inhibitors described above as well as the growing number of SIRT1 inhibitors ^69^. To inhibit late lytic replication, classical drugs such as valganciclovir can be used. Other possibilities include inducing RTA-independent lytic replication, possibly by targeting metabolic processes, where a significant decrease in viral progeny is observed. Continued advances in this field will provide additional insights into the biology and pathogenesis of KSHV infection as well as better treatments and cures for KSHV-related cancers.

## Abbreviations

DDR, DNA damage response; DE, delayed early; EndMT, endothelial-to-mesenchymal transition; HDAC, histone deacetylase; HSP90, Heat shock protein 90; IE, immediate early; KAP1, Krüppel-associated box domain-associated protein 1; KICS, KSHV inflammatory cytokine syndrome; KSHV, Kaposi’s sarcoma-associated herpesvirus; LANA, latency-associated nuclear antigen; MCD, multicentric Castleman’s disease; PEL, primary effusion lymphoma; RTA, replication and transcription activator; ROS, reactive oxygen species; SIRT, sirtuin; TLK, tousled-like kinase; TPA, 12-O-tetradecanoylphorbol-13-acetate; vIL-6, viral interleukin-6.
